# Insights into orthopedic and trauma emergency care and current challenges in Germany

**DOI:** 10.1007/s00068-025-02829-3

**Published:** 2025-04-22

**Authors:** Jonas Roos, Sophia Helm, Amadeo Touet, Davide Cucchi, Kristian Welle, Leonie Weinhold, Ingo Graeff, Martin Gathen

**Affiliations:** 1 Department of Orthopedics and Trauma Surgery, University Hospital of Bonn, Venusberg-Campus 1, 53127 Bonn, Germany; 2 Institute for Medical Biometrics, Informatics and Epidemiology, University Hospital of Bonn, Bonn, Germany; 3 Interdisciplinary Emergency Department (INZ), University Hospital of Bonn, Bonn, Germany

**Keywords:** Emergency department, Resource utilization, Manchester Triage System, Emergency care reform, Healthcare efficiency

## Abstract

**Background:**

The reform of emergency and acute care in Germany focuses on efficiently managing patient flows to reduce overcrowding in emergency departments, primarily caused by a rise in patients with low urgency and a lack of alternative care options. The aim of this work was to analyze the emergency care of orthopedic and trauma surgery patients in a Level I trauma center in order to improve patient care and manage resources more effectively.

**Material and methods:**

In this retrospective study, the data of orthopaedic and trauma surgery patients from the emergency department of a university in 2022 were analyzed. Data included demographics, triage levels, transport modes, diagnoses, and treatment outcomes. Process metrics like length of stay and time to medical contact were also evaluated. Descriptive and statistical analyses were conducted to examine patient distribution and resource use.

**Results:**

A total of 9414 patients (47.5% females; mean age 42.4 ± 24.1 years) were included, with 7500 treated for trauma and 1914 for orthopedic diagnoses. Most patients (79.6%) received outpatient care, while 21.3% were admitted as inpatients. Manchester Triage System distribution revealed 48.5% green, 31.0% yellow, 14.8% orange, and 0.8% red. The most common MTS flowcharts were limb problems (38.3%), falls (19.7%), and back pain (11.1%). Ambulance transport accounted for 33.7% of arrivals, while 65.1% presented independently. The average length of stay in the ED was 213 min, varying significantly across triage categories and working hours.

**Conclusion:**

This study highlights key challenges in orthopedic and trauma emergency care, emphasizing the prevalence of low-urgency cases contributing to overcrowding. Enhancing outpatient care capacity and implementing reforms like integrated emergency centers and optimized triage systems are crucial to improving efficiency and aligning resources with patient needs.

## Introduction

The Emergency Department (ED) is the primary point of contact for patients seeking short-term care in a hospital setting. In recent years, there has been an increase in presentations at the ED, leading to the problem of frequently overcrowded emergency rooms [1]. This poses the risk of delayed care in the event of urgent medical emergencies and places a high burden on the deployed staff. In particular, the number of patients with low urgency for treatment has increased [2]. The German federal government now aims to change this and reform emergency care [3].

Every individual patients accessing emergency care should undergo an assessment and classification process, allowing for the prioritization of those facing the most critical problems who need immediate care [4]. The increased patient influx results in extended waiting times for triage, prolonged duration of stay in the ED, patients leaving the emergency room prematurely without physician contact due to long waiting periods, and ultimately an escalation in medical errors [5–8]. Overcrowding appears to be particularly prevalent in EDs with over 40,000 visits per year [6].

The reasons for the increased number of patients are multifaceted. On one hand, an enhancement in the interface with emergency medical services, along with a growing number of walk-in patients, contributes to a rise in patient volumes [9]. Additionally, factors such as rising patient expectations and limited accessibility to outpatient care also play a significant role [10]. A considerable portion of patients in the ED are treated within the specialties of orthopedics and trauma surgery [11]. Among the most common reasons are back pain and lower extremity pain, which in many cases are not acute and have already been treated on an outpatient basis [12].

The aim of this study is to analyze and describe the care situation of orthopedic and trauma surgery patients in a Level I ED. It focuses on examining the reasons for patient visits, situations of overload, differences between self-referrals and medical referrals, as well as the health impacts on patients. This analysis is intended to provide insights and data for improving patient care and managing the department’s resources more effectively.

## Material and methods

### Study design

A retrospective analysis of patient volume in orthopedics and trauma surgery (O/T) at a university maximum care provider was conducted for the year 2022. The Hospital is a certified Level I trauma center (LV1 ED). The study was approved by the local institutional review board (No. 166/23).

### Inclusion and exclusion criteria

All patients accessing the LV1 ED of the investigation center between January 2022 and December 2022 were considered eligible for this study, including both self-referred patients, GP-referred patients and patients accessing by emergency services. Patients primary assigned to the O/T consultant after a nurse-based triage with direct specialty access were included in the study. Patients primarily triaged to a different specialty with secondary O/T consult and inpatients who were only treated in the emergency center for an interim intervention were excluded from the study.

### Standard operative procedures

On arrival at the ED, patients are triaged by a trained nurse who collects demographic data, takes vital signs and takes a brief medical history. Based on this initial assessment, patients are allocated to a specialist department. Patients are then triaged using the Manchester Triage System (MTS).

The MTS is a widely adopted method in the European Union for classifying the urgency and predicting the risk of patients in emergency care [13–15]. The MTS categorizes patients’ urgency for treatment into five levels based on their condition: red for immediate treatment, orange for very urgent, yellow for urgent, green for standard urgency, and blue for non-urgent. This system utilizes 53 pre-defined flowcharts, which align with the primary complaint reported by the patient [13, 16, 17]. In addition, there are defined clinical discriminators. This standardized approach ensures consistent prioritization of treatment and helps to efficiently allocate resources within the ED.

Following triage, patients are referred directly to a specialist. This direct referral reduces delays and ensures that patients receive targeted treatment as quickly as possible. The ED phase is completed as soon as the diagnosis has been established and the specialist has assigned an International Statistical Classification of Diseases (ICD) code. At this stage, patients are either discharged, admitted to hospital or referred for further outpatient treatment.

### Data extraction / collected variables

Patients’ demographic data, including age, gender, case status, type of transportation used, day and time of first contact (weekday), MTS, clinical discriminators, ICD codes, discharge destination (outpatient, inpatient, death in ED), process-related metrics (total length of ED-stay and relevant time intervals between key process steps) and health insurance were extracted from the local electronic data management system (ORBIS®, Dedalus Group, Mailand, I) and entered into a spreadsheet for analysis.

### Statistical analysis

Characteristics of the data are described using means with standard deviations (SD) for continuous variables and frequency distributions with percentages for categorical variables.

A generalized linear model with a gamma distribution was employed to investigate whether differences in the total length of stay (in minutes) were associated with triage levels and time of day. The time of day was categorized into two groups: periods when the majority of the patient’s stay occurred during regular working hours (Mon- Fri, 8am to 5 pm) or outside. All analyses were carried out using the R Software for Statistical Computing Version 4.4.0.

### Declaration of generative AI and AI-assisted technologies in the writing process

GPT-4o was used for language improvement and general manuscript revision. After using this tool, the authors reviewed and edited the content as needed and take full responsibility for the publication’s content.

## Results

### Descriptive

A total of 9414 patients (mean age 42.4 ± 24.1 years, 47.5% females) were included in the study. 7.500 (79,7%) patients were being treated for a trauma surgery diagnosis and 1.914 (20,3%) for an orthopedic diagnosis. 79.6% of the patients could be treated as outpatients and 21.3% were admitted as inpatients after treatment. 1% were treated in the emergency room during the inpatient stay. There was a uniform distribution of patients across the days of the week, with a slight increase on Monday (15.1%), Tuesday (14.3%), Wednesday (13.6%), Thursday (14.0%), Friday (14.6%), Saturday (14.3%) and Sunday (14%).

**Fig. 1:**
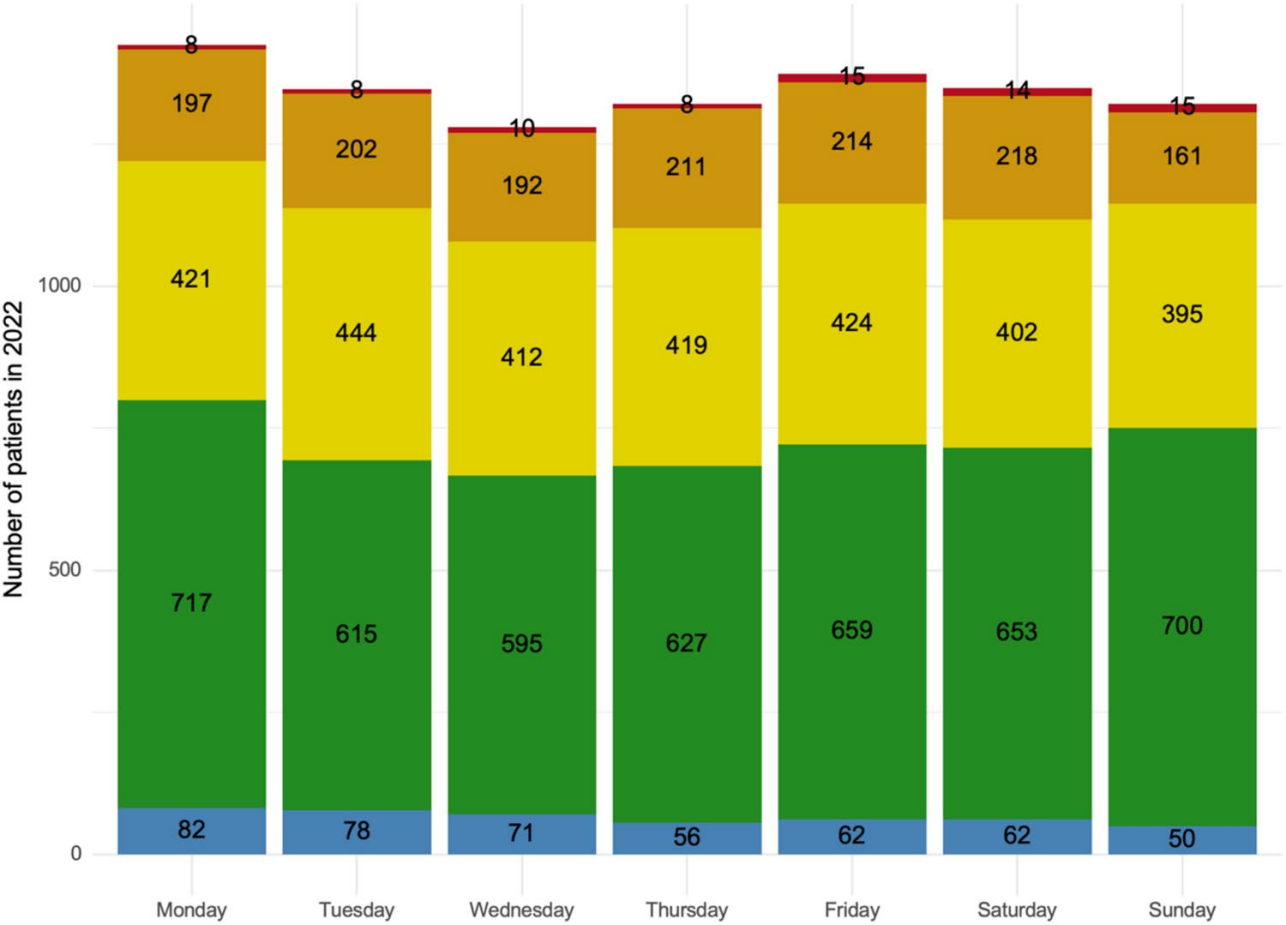
Weekly distribution of MTS triage categories (blue, green, yellow, orange, and red) by day of the week in 2022. The stacked bar chart represents the total number of patients treated each day, broken down by triage urgency, with green and yellow categories dominating across all days

According to the MTS classification, 461 (4.9%) were triaged blue, 4.566 (48.5%) green, 2.917 (31.0%) yellow, 1.395 (14.8%) and 78 (0.8%) red. A distribution of the MTS classification by day of the week is shown in Fig. 1.

### Transport

A total of 6.131 patients (65.1%) presented to the ED independently. Ambulance transport accounted for 2.217 patients (23.5%), 300 patients (3.2%) arrived via patient transport vehicles, 564 patients (6.0%) via emergency physician support, and 92 patients (1.0%) via air rescue services. For 114 patients (1.2%), this information was not collected.

### Flow Charts/Discriminators MTS

The most common MTS flowcharts were limb problems, accounting for 3.611 cases (38.3%), followed by falls with 1.859 cases (19.7%) and back pain with 1.050 cases (11.1%). Other frequently observed diagnoses included major trauma (463 cases, 4.9%), wounds (412 cases, 4.4%), and head injuries (385 cases, 4.1%). Less common categories were general unwell adult (332 cases, 3.5%), Assault (146 cases, 1.6%), general indicators (134 cases, 1.4%), and neck pain (130 cases, 1.4%). The remaining diagnoses were grouped under “Other” (895 cases, 9.5%), with one case missing data.

Regarding clinical discriminators, the most frequent was “younger problem” (2.555 cases, 27.1%), followed by “moderate pain” (1.654 cases, 17.6%) and “younger mild pain” (1.097 cases, 11.6%). Notable but less common indicators included noticeable injury mechanisms (758 cases, 8.0%), swelling (702 cases, 7.5%), and no specific indicator (461 cases, 4.9%). Rare indicators included unstoppable minor bleeding (349 cases, 3.7%), particular infection risks (230 cases, 2.4%), mismatched medical history (190 cases, 2.0%), and gross malalignment (168 cases, 1.8%). Additional less frequent indicators were categorized as “Other” (1.246 cases, 13.2%), with eight cases (0.1%) missing data.

Regarding triage and the type of transport to the emergency room, the following distribution resulted (see Table 1):

**Table 1:** Distribution of MTS triage categories by mode of patient transport to the ED

	MTS triage				
	Blue	Green	Yellow	Orange	Red
Ambulance transport	30	602	1.027	536	21
Emergency physician support	0	11	116	402	35
Patient transport	13	152	114	21	0
Air rescue services	0	7	13	55	17
Other	6	67	31	10	0
Independently	412	3.727	1.616	371	5

### ICD code

**Fig. 2:**
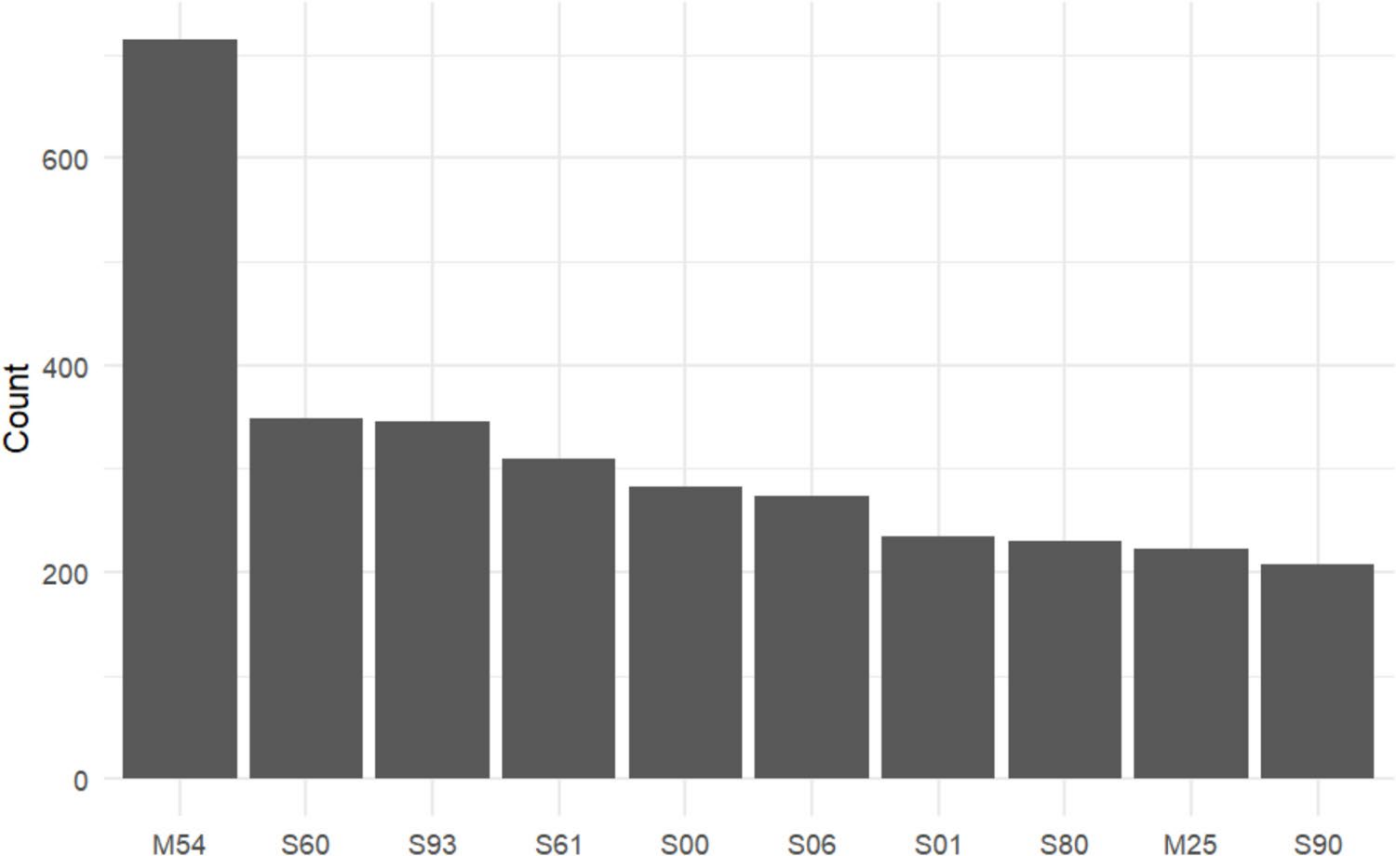
Analysis of ICD codes for outpatients with the most common diagnostic categories. Diagnosis categories include M54 (dorsalgia/back pain), S60 (superficial injury of the wrist and hand), S93 (dislocation, sprain and strain of joints and ligaments at ankle and foot level), S61 (open wound of wrist and hand), S00 (superficial injury of head), S06 (concussion), S01 (open wound of the head), S06 (skull injury), S80 (superficial injury of lower leg), M25 (other joint disorders)

A total of 2.714 distinct ICD codes were recorded, grouped into 830 main categories. Among these, 399 categories were documented more than three times, and 136 were recorded more than 20 times. Figure 2 and Table 2 shows an overview of the most common diagnoses of outpatients and inpatients.

**Table 2:** Analysis of ICD codes for inpatients with the most common diagnostic categories

ICD code combinations	Frequency
Z11	315
Z11, S06	151
Z11, S06, S01	32
Z11, I10	31
Z11, S72	26
Z11, S06, S00	24
Z11, B95	22
Z11, S22	21
Z11, S06, S02	18
Z11, S06, S02, S01	18

Diagnosis categories include Z11 (special screening for infectious diseases), S06 (concussion), S01 (open wound of the head), I10 (essential (primary) hypertension), S72 (fracture of the femur), S00 (superficial injury of head), B95 (Streptococcus and Staphylococcus as the cause of diseases classified to other chapters), S22 (fracture of rib(s), sternum and thoracic spine), S02 (fracture of the skull and facial bones), S01 (open wound of head)

### Process-related metrics

The overall average length of stay in the ED was 213 ± 148 min. The time from triage to first documented medical contact had a mean duration of 42.4 ± 54.8 min, though 2.9% of data points were missing. The interval from admission to first medical contact averaged 52.3 ± 58.9 min, 2.9% of values were missing.

The time from first medical contact to the end of treatment had a mean duration of 150 ± 140 min, while the period from the end of treatment to discharge was significantly shorter, with a mean of 12.2 ± 57.0 min. In each of these categories, approximately 2.9% of cases lacked complete data.

**Fig. 3:**
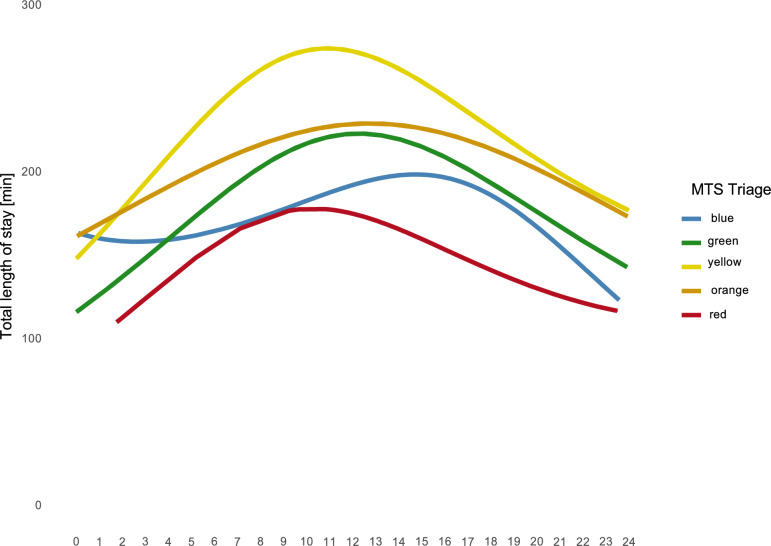
Total length of stay (in minutes) across different MTS triage categories (blue, green, yellow, orange, red) plotted against the time of patient arrival (X-axis) in the ED. Each line represents the average length of stay for patients within a specific triage category over 24 h

The total length of stay varied significantly across MTS triage categories throughout the day. Patients in the yellow category consistently had longer stays compared to other categories, peaking during midday hours (239 ± 159 min). Orange (214 ± 145 min) and (200 ± 137 min) categories demonstrated relatively stable stay durations, with minor fluctuations. Blue (181 ± 159 min) and red (160 ± 117 min) categories exhibited the shortest lengths of stay overall, with no significant peaks across the 24-h period (See Fig. 3).

**Fig. 4:**
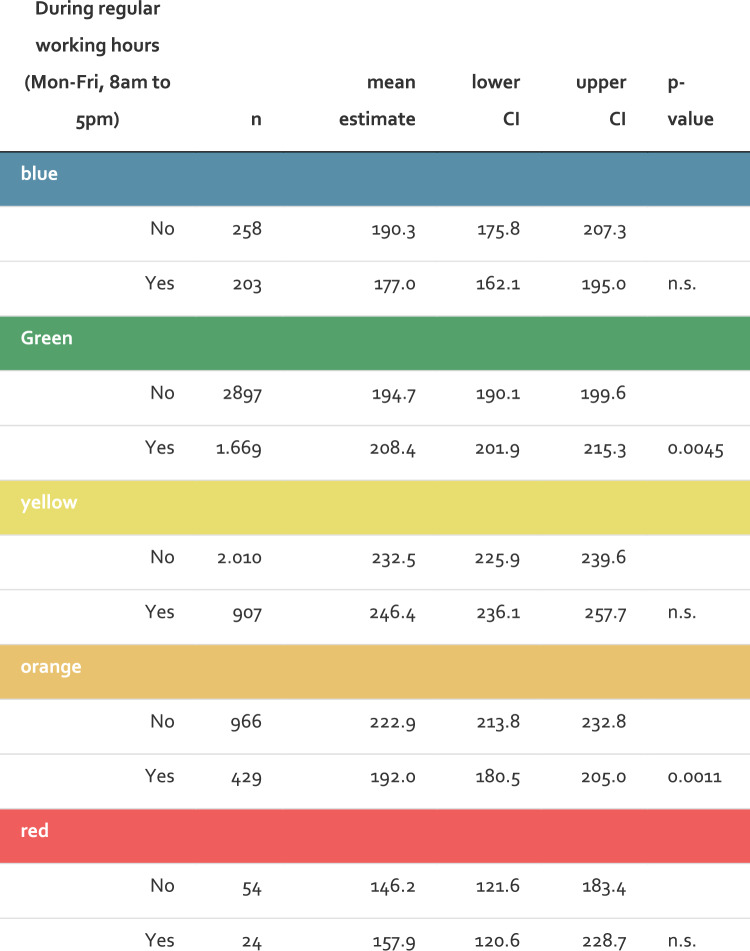
The figure illustrates the total length of stay in the ED across MTS triage categories, stratified by whether the patients presented during regular working hours (Monday to Friday, 8 am to 5 pm) or outside of those hours

For patients in the green category, the mean length of stay was significantly longer during working hours (208.4 min) compared to outside working hours (194.7 min), with a p-value of < 0.001. In contrast, for patients in the orange (very urgent) category, the mean length of stay was shorter during working hours (192.0 min) compared to outside working hours (222.9 min), with a p-value of < 0.01. For other triage categories, no significant differences in length of stay were observed between working and non-working hours (See Fig. 4).

The majority of patients were treated on an ambulatory basis (N = 7.309, 77.6%), with the most frequent MTS triage category being green (standard urgency, 55.6% ambulatory and 23.9% inpatient). Inpatient cases (N = 2.107, 22.4%) predominantly fell into the yellow (urgent, 37.5%) and orange (very urgent, 33.6%) categories. Only a small proportion of patients were triaged as red (immediate treatment, 0.8%), with most requiring inpatient care (82.1%) (see Fig. 4 and Table 3).

**Table 3:** Distribution of patients by MTS triage category and treatment status (ambulatory vs. inpatient)

MTS triage category	Ambulatory (N = 7309)	Inpatient (N = 2107)	Overall (N = 9416)
Red	12 (0.2%)	64 (3.0%)	78 (0.8%)
Orange	688 (9.4%)	707 (33.6%)	1.395 (14.8%)
Yellow	2.127 (29.1%)	790 (37.5%)	2.917 (31.0%)
Green	4.062 (55.6%)	503 (23.9%)	4.565 (48.5%)
Blue	420 (5.7%)	37 (1.8%)	461 (4.9%)

The table summarizes the absolute numbers and proportions of patients for each triage category, with inpatient cases combining pre- and post-inpatient stays

### Health insurance

The insurance categories can be consolidated into statutory health insurance (e.g., AOK, Barmer, Techniker Krankenkasse; 76.5%), private insurance (e.g., Allianz Private Krankenversicherung, Debeka, DKV; 13.4%), and occupational accident insurance (e.g., *Berufsgenossenschaften, Unfallkassen*; 10.0%). Among these, the three most frequent payers are Techniker Krankenkasse (1.448 cases, 17.2%), AOK Rheinland/Hamburg (1644 cases, 19.5%), and Barmer (442 cases, 5.2%).

## Discussion

This study provides valuable insights into the current state of orthopedic and trauma surgery emergency care in Germany in a maximum care hospital. Our findings highlight several key challenges for ED, including the need to optimize resources to better manage patient distribution, urgency levels and treatment times.

A majority of patients (77.6%) were treated on an ambulatory basis, with a predominance of green (90 min) and yellow (30 min) triage categories. This reflects the significant influx of low-to-moderate urgency cases that contribute to ED overcrowding, a trend previously reported in the literature [12]. Our analysis shows that there were longer treatment times for green and yellow triaged patients, particularly at midday, suggesting a temporary overload in the ED. However, as only orthopaedic and trauma surgery patients were examined in our analysis, it is not possible to draw a conclusion about the overall situation in the ED, meaning that the individual factors are not completely clear. This should be investigated in further studies.

The high percentage of inpatient admissions in the yellow and orange categories further emphasizes the critical role of EDs in managing patients with complex conditions requiring immediate care. Notably, red cases (immediate treatment) represented only 0.8% of the total but showed a heavy reliance on inpatient resources, aligning with the expected prioritization of critically ill patients. An interdisciplinary study on the age and gender distribution in the central ED showed a similar distribution in the Manchester triage level with leading yellow and green triaged patients and a very low proportion of red triaged patients overall. There were age-related differences with an increasing urgency in older people [18]. This was not considered separately in our study and should be taken into account in future analyses.

Analysis of treatment times revealed that patients in the green category had significantly longer stays during regular working hours than outside of these hours. This counterintuitive result may be attributed to increased patient volume or procedure bottlenecks during peak hours, despite better staffing. Conversely, patients in the orange category had shorter stays during working hours, suggesting that resource allocation for higher urgency cases may be more efficient. On average, patients stayed in the ED for 213 min. This compares to other work showing a comparable length of stay between 101 and 240 min [19–21]. These findings suggest opportunities to optimize workflow and triage processes, particularly for lower priority patients, to reduce delays and improve overall efficiency.

The variety of ICD codes included in this study reflects the complexity and wide range of conditions treated in the orthopedic and trauma ED. Outpatient cases were dominated by musculoskeletal injuries, such as dorsalgia (M54), knee sprains (S83), and wrist injuries (S63), many of which are common but non-critical presentations. This is also reflected in the established literature, where back pain and extremity problems were among the most common orthopedic presentations in the ED [22, 23]. While the treatment of extremity injuries often requires X-ray examinations, in the absence of clinical signs of serious pathology, diagnostic imaging or laboratory testing is often not necessary or patients receive X-ray images that are not indicated and patients can be treated in the outpatient setting [24, 25].In contrast, inpatient cases showed a higher prevalence of systemic or life-threatening conditions, such as femoral fractures (S72) and fractures of rib(s), sternum and thoracic spine (S22). The overlap of diagnostic categories, especially in inpatients, highlights the complexity of the cases treated and underscores the importance of multidisciplinary care. It should also be noted that inpatients were tested for COVID-19 during this period, which resulted in the ICD- code Z11. Nevertheless, a large proportion of patients from the lower triage levels are admitted to hospital. This could be due to the fact that the MTS tables are based on the symptoms reported by the patients and not on the underlying causes [17]. Consequently, patients with minor complaints or a longer medical history may be placed in lower triage levels. This applies, for example, to patients with wound healing complications following surgery who show no signs of sepsis and report minor symptoms but require surgery, or to patients with mild traumatic brain injury who are admitted for inpatient monitoring. Further sub-analyses in future studies are needed to better match the described symptoms with clinical relevance.

The analysis of insurance status showed a normal distribution of privately insured patients, with a slightly lower proportion of statutory insured patients [26]. Ten percent of the patients were covered by occupational accident insurance and had suffered a work-related injury. The high proportion of these patients is not unexpected given that the hospital in question is licensed by the employers’ liability insurance association. Previous literature reports a rate of 12%, which is consistent with our findings [27].

The results show that there is a significant reliance on transport assistance: 33.7% of patients are admitted by ambulance, emergency doctor or air ambulance. Despite this, 79.6% of cases are treated as outpatients and many ED treatments could potentially have been handled in the outpatient sector had the appropriate infrastructure been in place. This highlights the inefficiencies of the current system, where limited outpatient treatment options and resources can contribute to unnecessary hospital stays. Increasing outpatient capacity and reducing reliance on transport assistance for non-critical cases could optimize resource use and reduce pressure on emergency and inpatient services. Another solution to reduce numbers is to introduce preventative approaches that reduce the occurrence of emergencies or requests for medical assistance [28].

The aim of the reform of emergency and acute care in Germany is to ensure timely, high-quality and cost-effective emergency care for the population in line with demand [29, 30]. This goal is to be achieved through the establishment of integrated control centers (ILS) and integrated emergency centers (INZ), among other things. For the remaining people seeking help, adequate care is to be ensured by the outpatient clinics of the Associations of Statutory Health Insurance Physicians (*KV-Praxen*) in regular and 24-h operation in order to better fulfill the care mandate of the KV clinics [30]. Additionally, INZ locations will incorporate both hospital emergency units and *KV* on-call practices, with centralized triage units guiding patients to the appropriate care level. Special centers for pediatric emergencies may also be established, supported by telemedicine to ensure comprehensive care for children and adolescents [31].

The findings of this study align with the goals of Germany’s emergency care reform, which seeks to streamline patient pathways and reduce ED overcrowding. The high volume of ambulatory cases classified as green or yellow supports the need for alternative care pathways. Moreover, the significant length of stay differences by triage category and time of day suggest that targeted interventions, such as demand-based staffing or triage automation, could improve efficiency and reduce waiting times. In addition to these aspects, the reform must also take into account the inadequate financing of hospital services, including the costs of digital initial assessment procedures, the establishment of Integrated Emergency Centers and necessary investments [32].

### Limitations and future directions

While this study provides a comprehensive overview of orthopedic and trauma emergency care at a single institution, its retrospective design may limit the generalizability of findings to other settings. Additionally, not all ED patients were studied, limiting a complete analysis of overcrowding. Also, it was not recorded whether the patients were self-referred patients and those referred by physicians. This limits the analysis of the need for treatment. Furthermore, the reliance on electronic medical records introduces potential biases related to data completeness and accuracy. Future studies should explore multicenter data and incorporate prospective analyses to validate these findings and develop evidence-based strategies for improving emergency care delivery.

## Conclusion

This study highlights the high proportion of low-urgency cases and ambulatory treatments in orthopedic and trauma emergency care, contributing significantly to ED overcrowding. Length of stay analysis revealed inefficiencies in managing low-priority cases during working hours, while critical patients were effectively prioritized. The diverse range of ICD-coded conditions underscores the complexity of cases, emphasizing the need for accurate triage and multidisciplinary approaches. Strengthening outpatient care and developing alternative pathways could optimize resource use and improve emergency care delivery.

## Data Availability

No datasets were generated or analysed during the current study.
